# Screening and Isolation of Bacterial Strains Able to Degrade Trimethylamine

**DOI:** 10.3390/microorganisms13061369

**Published:** 2025-06-12

**Authors:** Sebastião V. T. F. de Almeida, Kilian Neves, Carla C. C. R. de Carvalho

**Affiliations:** 1 Department of Bioengineering, iBB-Institute for Bioengineering and Biosciences, Instituto Superior Técnico, Universidade de Lisboa, 1049-001 Lisbon, Portugal; sebastiao.tavares@tecnico.ulisboa.pt (S.V.T.F.d.A.); kilian.neves@gmail.com (K.N.); 2 Associate Laboratory I4HB—Institute for Health and Bioeconomy, Instituto Superior Técnico, Universidade de Lisboa, 1049-001 Lisbon, Portugal

**Keywords:** fishy smell, volatile organic compounds, methylamine, bacteria, microalgae

## Abstract

Methylamines are present in numerous organisms and microorganisms capable of de novo trimethylamine (TMA) production are widely distributed, including microalgae. However, such compounds may hamper the application of microalgae biomass in commercially interesting products, such as food and feed products, due to the strong fishy smell. In the present study, several bacteria able to degrade TMA were isolated. Among them, a *Staphylococcus saprophyticus* strain was found particularly suitable to degrade TMA. After finding the best culture conditions, a bioprocess system was developed allowing the degradation of TMA from microalgae in a reactor by *S. saprophyticus* cells present in a second reactor without direct contact with media from both reactors. The system was found to be limited by TMA transfer through the gas phase, with the cells being able to degrade all available TMA.

## 1. Introduction

Fish and microalgae are two essential elements of the marine ecosystem and of the human diet, both making substantial contributions to global nutrition. Microalgae, single-cell photosynthetic organisms, are renowned for their high nutrient content, including proteins, lipids, and essential vitamins, making them a valuable resource for food, feed, and biofuel production [1,2,3,4]. Despite the nutritional value of fish and microalgae, their components such as proteins, carbohydrates, and fats, may decompose during storage, producing volatile organic compounds known for their malodour [5]. One of these compounds is trimethylamine (TMA) and its accumulation can lead to a strong, off-putting odour, that may reduce their marketability and acceptability [6].

The high concentration of TMA in microalgae poses a problem for their use in food and feed industries, where sensory qualities are crucial. TMA can negatively impact the acceptability of microalgal products, limiting their commercial potential and value. Furthermore, TMA is a volatile organic compound that has a low odour threshold concentration (0.2 μg m^−3^; TMA odour threshold between 0.00021 ppm and 0.00058 ppm can be found in the literature), and it has been identified as potentially toxic and likely carcinogenic [7,8,9].

TMA and other methylamines in seawater and sediments may derive from marine biota or from the degradation of compounds containing nitrogen, such as glycine betaine, trimethylamine-*N*-oxide (TMAO), and choline, and are important carbon, nitrogen, and energy sources for diverse microorganisms [10,11]. TMA is frequently found in wastewater treatment plants, and in effluents from fishmeal processing plants and livestock farms, causing health issues in humans such as headaches, nausea, and eye and respiratory system irritation at a concentration of 15 ppm [12,13]. In order to prevent risks to safety and health, as well as to lessen the effects on the environment (such as eutrophication, acid rain, and the greenhouse effect), and to increase the market acceptability of microalgae, it is imperative that TMA-laden emissions be properly managed in accordance with environmental regulatory limits [14].

When it comes to reducing toxic and unpleasant volatiles, biotechnology has demonstrated to be more affordable and environmentally friendly than physico-chemical methods [15,16]. Thus, to address this problem, innovative solutions have involved the use of marine bacteria capable of using TMA as a carbon source. Given the prevalence of TMA in marine habitats, marine bacteria that can use TMA as their only source of carbon and/or nitrogen [17], or their enzymes [18], may offer a solution to the malodour problem. In fish and other marine biota, TMA accumulates after death as a result of conversion of the oxygenated odourless precursor, trimethylamine *N*-oxide (TMAO), which is an effective osmolyte [18,19]. TMAO is thought to be especially prevalent in organisms found in the deep ocean since it functions as an osmolyte, stabilising proteins under environmental stressors like osmotic pressure [19,20]. When the hadal snailfish *Notoliparis kermadecensis*, the world’s second-deepest known fish, was captured from 7000 m deep, it was found that this fish contains the highest recorded TMAO content [20].

Certain marine bacteria possess the enzymatic machinery to degrade TMA, converting it into less volatile and non-odorous compounds like TMAO. This oxidation of TMA into TMAO is catalysed by monooxygenases, which are enzymes that catalyse the insertion of oxygen into their substrates [18]. In this case, NADPH is used as a cofactor during reaction [18,21]. These enzymes have been discovered to be prevalent in marine bacterial metagenomes [19,22]. The first member of the flavin-containing monooxygenase to be characterised in bacteria was described in the marine strain *Methylophaga aminisulfidivorans* SK1, which belongs to Gammaproteobacteria [23]. TMA monooxygenases have also been described in several Alphaproteobacteria, including *Roseovarius nubinhibens*, *Methylocella silvestris* BL2, *Roseovarius* sp. strain 217, *Ruegeria pomeroyi* DSS-3, and *Pelagibacter ubique* strains HTCC1002 and HTCC721 [22,24]. These enzymes have also been discovered in bacteria from different environments: while flavin-containing monooxygenase producer *Corynebacterium glutamicum* was isolated from soil [25], *Nitrincola lacisaponensis* was isolated from an alkaline saline lake [26]. The main advantage of using whole cells in comparison to using enzymes is that cells can naturally regenerate cofactors [27], in this case, NADPH, during the transformation of TMA into TMAO.

The aim of the present study was to isolate and identify a marine bacterium that could use TMA as a carbon and/or nitrogen source in order to eliminate this malodour compound from microalgal cultures and aquaculture systems. This could also improve the sensory qualities of microalgal products.

## 2. Materials and Methods

### 2.1. Sampling Collection and Bacterium Isolation

Enrichment cultures with samples collected in Samouco salterns (GPS: 38.7352542, –8.9981561; 38°44′06.9” N 8°59′53.4” W), Alcochete, Portugal, were carried out with 1% (*v*/*v*) TMA (from TCI, Tokyo, Japan; sold as a solution of 28% in water, ca. 4.3 mol/L) as sole carbon and energy source. Isolation of strains on agar plates was made after 2 weeks, and after 4 and 8 months of enrichment cultures, using plates with marine agar (MA; Condalab, Madrid, Spain) and tryptic soy agar (TSA; BBL^TM^ trypticase^TM^ soy agar from BD). A volume of 50 µL of culture was spread per plate and three plates were made per enrichment culture. The plates were incubated at 30 °C and observed after 24 and 48 h.

All colonies that could be observed, after 48 h, with the naked eye, were used to inoculate new MA and TSA plates to allow for their isolation and characterisation. The initial plates were kept at 30 °C for up to 7 days to confirm that no other colonies developed.

The isolates are maintained at −80 °C at the laboratories of the iBB-Institute for Bioengineering and Biosciences, Lisbon, Portugal.

### 2.2. Bacterial Identification

The Sherlock^®^ Microbial ID System from MIDI, Inc. (Newark, DE, USA) was used to initially identify the isolates by their lipid profile. Briefly, the cells were grown on tryptic soy agar (TSA; from Fluka, Buchs, Switzerland) at 30 °C and harvested after 24 ± 1 h. After harvesting, the fatty acids (FAs) of the isolates were extracted and simultaneously methylated to fatty acid methyl esters (FAMEs), using the Instant FAME method from MIDI, as previously described [28,29]. FAMEs were analysed by gas chromatography using an Agilent Technologies 6890N gas chromatograph (Agilent, Santa Clara, CA, USA), with a flame detector and a 7683 B series injector. The column used was a 25 m Agilent J&W Ultra 2 capillary column. Strain identification was performed by comparing the FAME profile of the cells with those in the database of the Sherlock^®^ software package (version 6.2), using the ITSA method.

The extraction of DNA from colonies of each isolate was carried out using the DNeasy Powerwater Kit (Quiagen GmbH, Hilden, Germany). The purification of DNA, PCR amplification of the segment of the 16S rRNA gene containing the variable regions V1-V9, and generation of the consensus sequence were performed by Stab Vida (Caparica, Portugal). BLAST analysis of the consensus sequence was performed using the National Center for Biotechnology Information website (https://blast.ncbi.nlm.nih.gov/Blast.cgi; accessed on 4 August 2023 and repeated on 27 February 2025).

### 2.3. Bacterial Growth

Cultures of the selected bacterium were grown initially on 24 well plates to assess the medium composition and the effect of TMA concentration on the growth rate. The plates, containing mineral medium [30] with 5 g/L NaCl, were closed after inoculation and the addition of TMA as a single carbon and energy source, with a crystal-clear breathable sealing tape (Breathe-Easy^®^ sealing membrane, from Sigma-Aldrich, St. Louis, MO, USA) to prevent TMA evaporation.

After finding the best initial conditions in microtiter plates, the effect of culture conditions, including media composition, TMA concentration, salinity, and nitrogen source composition, on cell growth was determined in cylindrical 250 mL closed flasks. These flasks contained 20 mL of culture medium and allowed sampling through an injection port without loss of liquid or TMA from the gas phase. Media composition tested included: mineral medium with TMA at different concentrations as sole carbon and energy sources; tryptic soy broth (TSB; BBL^TM^ trypticase^TM^ soy broth from BD); and marine broth (MB; Condalab, Madrid, Spain). As a nitrogen source, meat peptone (Merck, Darmstadt, Germany) was initially used and compared with other nitrogen sources, such as ammonium sulphate and ammonium nitrate, using equivalent concentrations of nitrogen.

The cultures were incubated at 30 °C and 200 rpm in an Agitorb 200 incubator (Aralab, Portugal). Samples were taken during culture time using sterilised 1 mL syringes, and biomass growth was monitored by optical density measurements at 600 nm in a UH5300 Spectrophotometer (from Hitachi, Tokyo, Japan).

### 2.4. TMA Quantification

TMA was extracted from 1 mL samples using 0.3 mL methyl *tert*-butyl ether (MTBE; Sigma-Aldrich) and quantified by gas chromatography in an Agilent 7820A gas chromatograph equipped with a 7693A autoinjector, and an Agilent 5977E quadrupole MS detector (all from Agilent Technologies, Santa Clara, CA, USA). An Agilent J&W Ultra-2 capillary column, working at a constant flow of 1 mL/min, allowed for peak separation. The GC injector was set at 200 °C, the MS source at 230 °C, the MS quad at 150 °C, and the MSD transfer line at 280 °C. The separation of products was achieved by programming the oven to an initial temperature of 40 °C and increasing the temperature to 240 °C at 15 °C/min. Peak identification was performed by comparison to the NIST mass spectral library version 2.2 using the Qualitative Analysis B.07.00 software, part of the MassHunter Workstation from Agilent, and by injecting commercial TMA (from TCI, Tokyo, Japan). Quantification of TMA was performed by the Quantitative Analysis B.07.00 software, which is part of the same MassHunter Workstation, after the preparation of a calibration curve.

All assays were performed at least in duplicate. The results are presented as average ± standard deviation.

### 2.5. Bioreactor System

Duran^®^ 500 mL GLS80 stirred reactors (from Duran Wheaton Kimble, Mainz, Germany) were used for the scale-up of the system. Air was supplied through an air pump EHEIM air 200 at 300 mL/min through a marprene tubing (from Watson-Marlow Fluid Technology Solutions, Wilmington, NC, USA) to the first reactor containing microalgal biomass suspended in deionised water. The end of the tube was immersed in the microalgae suspension so that air was bubbled through the liquid. The air enriched in TMA was transported to the second reactor through a marprene tubing and bubbled through the bacterial cell suspension in a mineral medium supplemented with 5 g/L peptone and 35 g/L NaCl. The air from the outlet of this reactor was bubbled in deionised water on a third reactor to assess the presence of TMA. The system worked at 30 °C and the reactors were magnetically stirred at 100 rpm (by magnetic stirrers from Heidolph, Schwabach, Germany).

## 3. Results and Discussion

### 3.1. Isolation of Strains Able to Convert TMA

Enrichment cultures with samples collected in Samouco salterns, Alcochete, Portugal, were carried out with 1% (*v*/*v*) TMA as the sole carbon and energy source. The sampling site was chosen because of the high concentration of algae and Archaea, which are known to produce TMA. Isolation of strains by spreading samples from the enrichment cultures on agar plates containing TMA as a carbon source was performed after 2 weeks, and 4 and 8 months of cultivation. After 2 weeks, 22 strains could be isolated, and in total, 53 strains could be isolated from the samples after spread plating in the three time periods. Around 67% of the isolates initially grew in TSA. When a colony was observed in MA, it was transferred to TSA for identification by analysis of the lipid profile. However, 10 isolates could not be grown on plates containing TSA.

**Figure 1:**
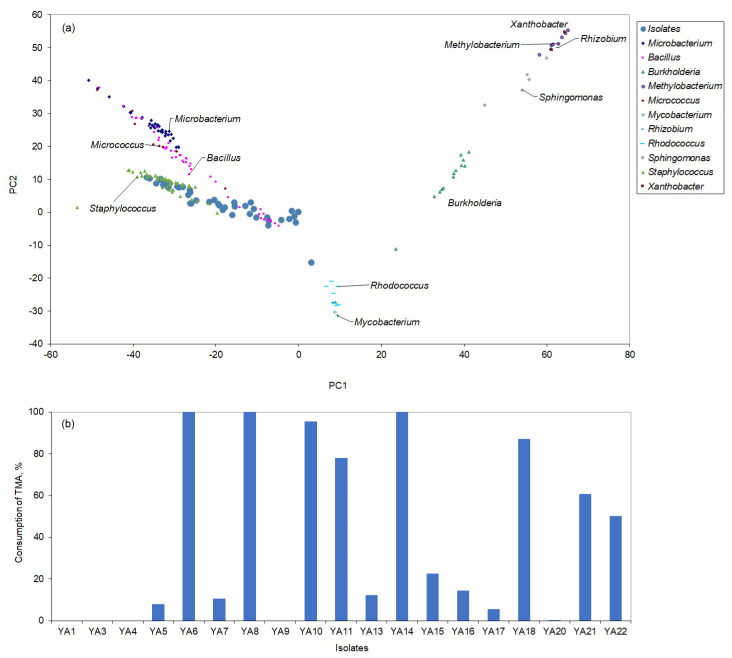
(**a**) Scores of the sampled isolates along the first two axes resulting from the Principal Components Analysis of the fatty acid composition of selected bacterial genera present in the Sherlock^®^ MIS library. (**b**) Percentage of TMA consumed in 48 h by selected isolates.

Using the fatty acid profile of the cells, it was possible to make an initial identification of isolates by comparing the profile of the cells to those of the species in the database of the Sherlock^®^ Microbial Identification system from MIDI, Inc. (Newark, DE, USA), as previously described [31]. It was also possible to compare the proximity of the isolates to the species in the database, even when no match was found by representing the isolates in the space defined by the first two principal components (PC) of the analysis, which represent the highest variance of the data (Figure 1a). Several isolates showed close proximity to the *Staphylococcus* genus.

After discarding isolates that grew slowly, the remaining isolates were evaluated for their ability to degrade TMA (Figure 1b). Isolates YA6, YA8, YA10, YA11 and YA14 could degrade over 95% of the TMA added to the reaction media in 48 h. 16S rDNA sequencing identified YA6 as *Staphylococcus equorum*, YA8 as *Sporosarcina psychrophila,* YA10 as *Staphylococcus saprophyticus*, and YA11 as *Staphylococcus cohnii*. YA14 could not be identified. Since YA10 was also found able to convert dicyclopentadiene, which is known for an unpleasant camphor-like odour, the strain was selected for further studies.

**Figure 2:**
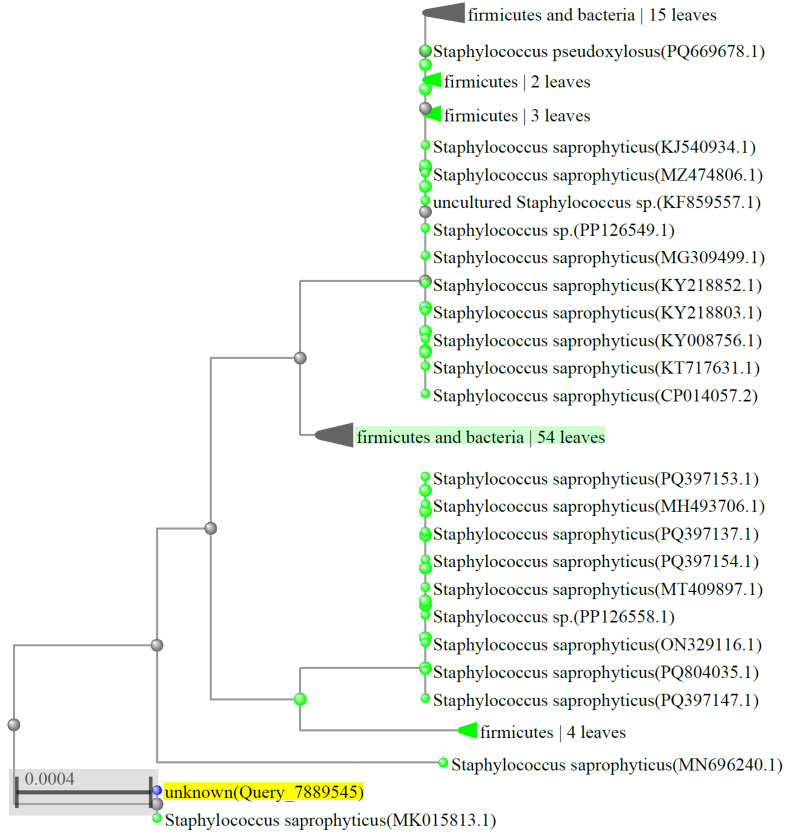
Molecular phylogenetic analysis with neighbour-joining method, representing a comparison between the 16S rRNA coding gene sequence of isolate YA10, highlighted in yellow, and those of species in the BLAST database for a maximum sequence difference of 0.5. The taxonomic name and sequence identification number are indicated for each sequence.

Sequencing of the 16S rRNA of YA10 and BLAST analysis of the consensus sequence showed that the isolate presents over 99.8% similarity to sequences of several *S. saprophyticus* strains (Figure 2).

### 3.2. Growth Conditions with Trimethylamine as a Carbon Source

#### 3.2.1. Effect of the Medium Used to Grow the Inoculum

It has long been known that the number and type of cells in the inoculum affect the growth of the main culture, especially in terms of the duration of the *lag* phase and the rate of subsequent exponential growth [32,33]. In the present study, the effect of the medium composition used for growing the inoculum on the maximum growth rate of *S. saprophyticus* cultures in a mineral medium with TMA as the sole carbon and energy source was assessed. The results were compared with cultures inoculated with cells that were washed to remove all nutrients from the inoculum culture.

**Figure 3:**
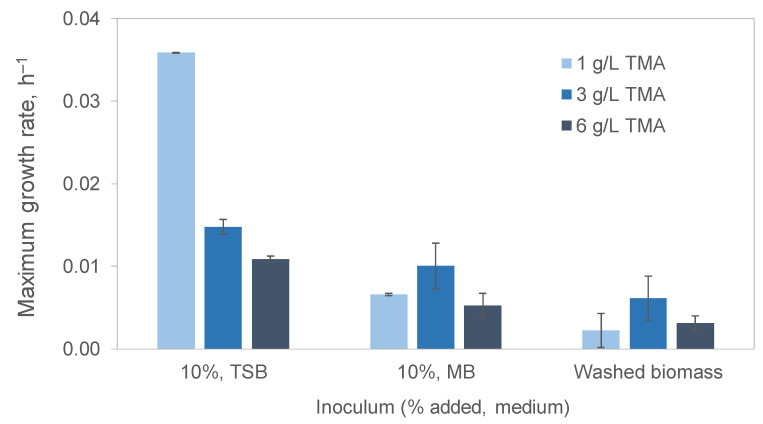
Maximum growth rate of *S. saprophyticus* cells on mineral medium containing 1, 3 and 6 g/L TMA when inoculated with 10% (*v*/*v*) of inoculum grown on TSB, MB or with washed cells that had grown in TSB.

The highest growth rate, µ_max_, achieved was 0.04 h^−1^ when the cells grew on 1 g/L of TMA in a mineral medium inoculated with 10% volume of a culture grown in tryptic soy broth (TSB; Figure 3). The µ_max_ was 5.4-fold higher than when the culture was inoculated with marine broth (MB) and 16-fold higher than when the cells grown on TSB were washed before being used as inoculum. This indicates that the 10% (*v*/*v*) of TSB that was added with the inoculum must have provided enough nutrients to help the cells grow on 1 g/L of TMA. However, TMA inhibited cell growth, and the µ_max_ decreased 2.4- and 11.5-fold when the cultures inoculated with 10% of the volume of cultures grown on TSB were maintained on 3 and 6 g/L TMA, respectively (Figure 3). Curiously, cultures inoculated with MB-grown cells and with cells grown on TSB but washed prior to inoculation grew faster with 3 g/L TMA than with 1 or 6 g/L.

The difference between TSB and MB composition is the following: NaCl is at a concentration of 5 g/L in TSB and 19.4 g/L in MB; the peptone concentration is 20 g/L in TSB and 5 g/L in MB; TSB has glucose at 2.5 g/L, whilst MB has several compounds such as sodium fluoride, sodium silicate and strontium chloride that are not present in TSB. Taking into consideration the sampling site, one would expect the *S. saprophyticus* cells to prefer the MB, even if diluted 10 times (only 10% of the volume corresponded to the medium brought with the inoculum). Nevertheless, when the inoculum corresponded to washed biomass, much lower growth rates were attained. This suggests that the 10% (*v*/*v*) of TSB and MB was sufficient to favour cell growth.

#### 3.2.2. Effect of Temperature, Stirring Speed and Salinity

**Figure 4:**
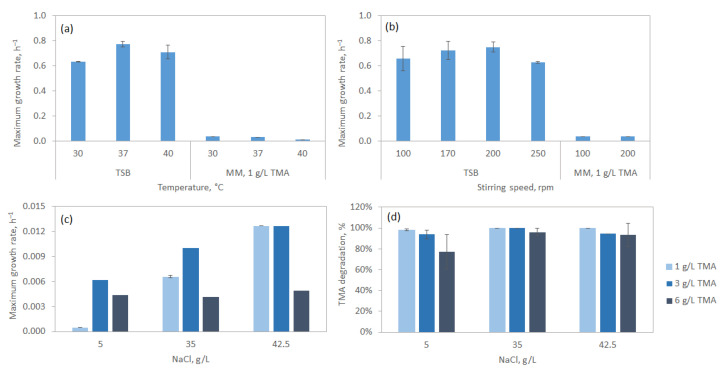
Influence of temperature (**a**) and stirring speed (**b**) on the maximum growth rate of *S. saprophyticus* cells in TSB and in MM containing 1 g/L TMA. The media contained 35 g/L NaCl. Effect of salinity on the maximum growth rate (**c**) and TMA degradation (**d**) of *S. saprophyticus* cells growing in mineral medium with 1, 3 and 6 g/L TMA.

When *S. saprophyticus* cells grew at different temperatures in TSB or in mineral medium with TMA, it was found that the cells grew fastest at 37 °C on TSB (µ_max_ was 0.77 h^−1^; Figure 4a). However, cells growing in a mineral medium with 1 g/L TMA grew faster at 30 °C (µ_max_ was 0.04 h^−1^) than at 37 or 40 °C. The vapour pressure of trimethylamine increases from 1946 mm Hg at 30 °C to 2621 mm Hg at 40 °C [34], which may explain why the growth of *S. saprophyticus* was faster at 30 °C when growing on TMA. The much richer composition of TSB, in comparison to the defined mineral medium with TMA as the carbon source, could explain the 21.5-fold increase observed between the µ_max_ in these two media (Figure 4a).

Regarding stirring speed during culture growth, the cells grew faster at 200 rpm (Figure 4b), regardless of the culture media. Nevertheless, the differences in µ_max_ observed between 100 and 200 rpm were within the standard deviation for both media.

The *S. saprophyticus* strain was isolated from a sample collected in a saltern where salt crystals could be observed by the naked eye and so the effect of NaCl concentration on the maximum growth rate was evaluated. In a mineral medium with 1 g/L TMA, a linear increase in µ_max_ with increasing concentration of salt was observed, with cells growing ca. 27 times faster in 42.5 g/L NaCl than in 5 g/L (Figure 4c). When 3 g/L TMA was used as a carbon source, an increase in µ_max_ with increasing concentrations of salt was also observed, and for the same NaCl concentration, the cells grew faster than with 1 g/L TMA. Curiously, a difference of only 11.6% was observed between µ_max_ observed at 5 and 42.5 g/L NaCl. Overall, the results suggest that a concentration of at least 35 g/L, which is the concentration of salt in seawater, favours the growth of *S. saprophyticus* in TMA. Concentrations of 50 and 70 g/L NaCl were also tested, but the bacteria formed large clusters of cells, which are signs of stress.

TMA degradation was also monitored after 3 days of cultivation when 1, 3 and 6 g/L TMA were used in media with NaCl concentrations of 5, 35 and 42.5 g/L (Figure 4d). With culture medium containing an initial concentration of 1 g/L TMA, *S. saprophyticus* consumed all available TMA when the concentration of NaCl was 35 and 42.5 g/L and consumed 98.2% of the initial TMA in the presence of 5 g/L NaCl. Maximum consumption of TMA in cultures with 3 and 6 g/L TMA was reached at 35 g/L NaCl, attaining 99.9 and 95.9% of the initial TMA, respectively. Since no TMAO was found in the analyses, TMA was used for cell growth and, thus, to produce biomass. The results suggest that *S. saprophyticus* did not convert TMA into TMAO but did degrade TMA.

#### 3.2.3. Nitrogen Source

**Figure 5:**
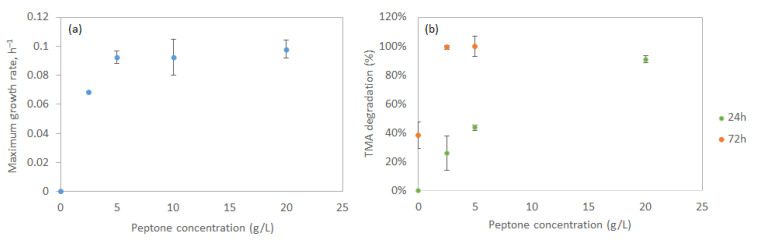
Effect of peptone concentration on the maximum growth rate of *S. saprophyticus* cells (**a**), and on the degradation of 1 g/L TMA observed after 24 and 72 h of growth (**b**).

To improve the growth of *S. saprophyticus* cells in a mineral medium, the addition of meat peptone was tested. It was previously suggested that the elimination of TMA by lactic acid bacteria could be favoured in a medium with peptone [35]. In the present study, the effect of meat peptone concentration in a mineral medium with 1 g/L TMA on the growth of *S. saprophyticus* was studied. A steep increase in the µ_max_ could be observed with increasing concentrations of peptone up to 5 g/L, but a plateau was reached for higher peptone concentrations at ca. 0.9 h^−1^ (Figure 5a).

Apart from peptone, different nitrogen sources, such as ammonium sulphate and ammonium nitrate, were also tested. Maintaining the peptone concentration at 10 g/L, the equivalent concentrations of nitrogen are 1.86 g/L of ammonium nitrate and 3.06 g/L of ammonium sulphate. However, *S. saprophyticus* grew much slower with those nitrogen sources at these concentrations than with meat peptone.

The effect of peptone concentration on TMA degradation was assessed after 24 and 72 h of *S. saprophyticus* growth in a mineral medium containing 1 g/L TMA (Figure 5b). After 24 h of culture, an increase in TMA degradation was observed with increasing concentration of peptone, reaching 91% TMA degradation at 20 g/L peptone. The final TMA concentration was at 0.0158 g/L, which is still above the odour threshold concentration of 0.2 μg m^−3^. However, after 72 h of growth, 99.1% of TMA had been degraded in the culture with 2.5 g/L peptone, with the remaining amount of TMA (6.1 mg/L) still above the odour threshold. At 5 g/L peptone, when µ_max_ was reached, the cells were able to degrade all TMA added to the culture medium in 72 h (Figure 5b).

To summarise, the conditions tested that allowed both the highest growth rate and TMA consumption were 30 °C, 200 rpm, 42.5 g/L NaCl, and 5–10 g/L peptone (Table 1).

### 3.3. Application of Trimethylamine Degradation in Bioreactor

**Figure 6:**
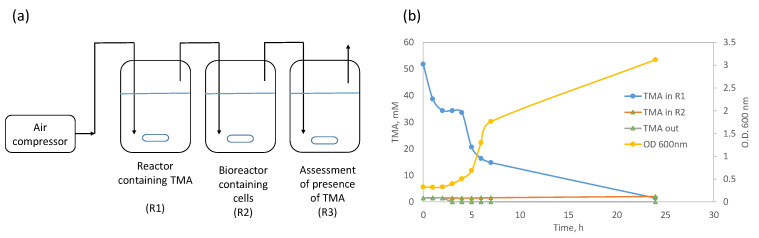
(**a**) Scheme of the reactor setup used to transfer, through the air stream, TMA from a liquid phase inside a reactor to a bioreactor containing *S. saprophyticus* cells growing in mineral medium supplemented with 5 g/L meat peptone, using TMA as a carbon source. (**b**) TMA concentration and optical density (O.D. 600 nm) of the cell culture in reactor R2.

TMA has a solubility in water of 890 g/L at 30 °C, and the measured value for Henry’s constant was reported to be 8.9 ± 0.1 mol L^−1^ atm^−1^ [36]. Henry’s law relates the solubility of a gas to its partial pressure above the liquid, explaining the gas/liquid partitioning of volatile compounds. In the previous sections, the cultures were maintained in closed flaks to prevent TMA losses through the gas phase. However, due to the high volatility of TMA, the possibility of adding this compound to the bacterial culture through the air phase was studied in bioreactors. Under the set-up tested, air at 1 vvm was pumped through a stirred reactor containing mineral medium and TMA (Figure 6a). The TMA was continuously transferred by the air from the first reactor to a stirred bioreactor containing *S. saprophyticus* cells. After leaving this latter bioreactor, the air was bubbled through a mineral medium in a third-stirred reactor to assess TMA consumption by the cells.

TMA concentration decreased 33.6% in the first reactor during the first 2 h (Figure 6b). This allowed for the onset of the exponential growth phase of the *S. saprophyticus* culture. The cells were growing in a mineral medium supplemented with 5 g/L of meat peptone and using TMA as a carbon source, reaching an optical density (O.D.) at 600 nm of 3.1, after 24 h of growth. This was sufficient to consume all TMA that had been transferred from the first reactor to the bioreactor where they were growing. In fact, the amount of TMA in the cultivation medium was always very low and reached zero at 24 h. Additionally, the TMA concentration remained zero after the initial 3 h in the last reactor, indicating that the cells consumed the TMA available in the bioreactor. The uptake of TMA by the cells should have been equal to or higher than the rate of TMA transfer from the first reactor to the reactor containing the cells, thus limiting the system by mass transfer.

**Figure 7:**
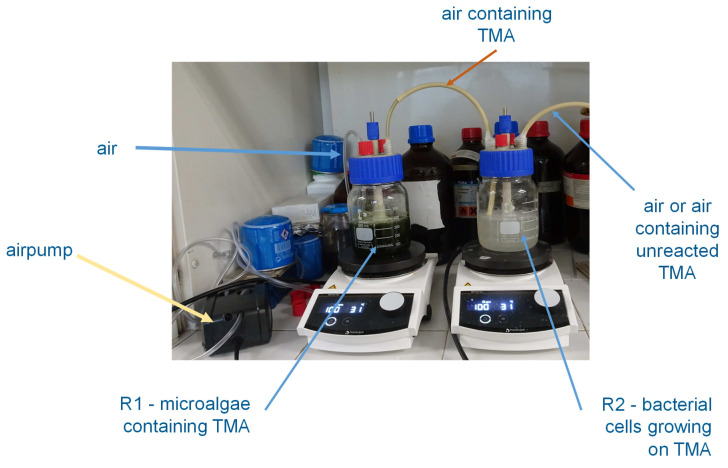
Reactor system with TMA being extracted from the microalgal biomass (left reactor) to the gas phase fed to the bioreactor containing growing *S. saprophyticus* cells (right). Air was injected by an air pump and transported through tubing with low permeability to air and wide chemical resistance to prevent TMA losses.

The fact that the bacterial cells and/or their enzymes do not contact the microalgal biomass (Figure 7) facilitates the application of the treated microalgae in food and/or feed products. We are currently preparing a manuscript detailing the changes in organoleptic properties of commercial microalgae after treatment with bacterial cells and/or enzymes.

## 4. Conclusions

The incorporation of microalgae in food products is dependent on their organoleptic characteristics, which highly influence consumer acceptance. In the present study, isolates efficient in degrading TMA were obtained by enrichment cultures of samples collected in a salt evaporation pond in a saltern. Among them, *S. saprophyticus* was found able to degrade TMA under industrially relevant conditions. The inexistence of TMAO in the analysis suggests that this strain degrades TMA and does not simply convert it.

A system transferring TMA from microalgal biomass contained in a bioreactor, through the gas phase, to a bioreactor containing *S. saprophyticus* cells, demonstrated the possibility of using these bacterial cells to degrade TMA from, e.g., microalgae biomass without direct contact between both cultures. The use of bacteria supports the principles of green technology and may offer a safe and eco-friendly solution to a prevalent problem in using marine biomass, such as microalgae and macroalgae.

## Figures and Tables

**Table 1:** Effect of initial TMA concentration, temperature, stirring speed, salinity and peptone concentration on the growth rate and TMA degradation ability of *S. saprophyticus* cells.

TMA (g/L)	Temperature (°C)	Agitation (rpm)	Salinity (g/L)	Peptone (g/L)	µmax, (h^−1^)	TMA Degradation (%)
1	30	100	35	5	0.04	-
1	30	100	35	5	0.04	-
1	37	100	35	5	0.03	-
1	40	100	35	5	0.01	-
1	30	200	5	0	0.0005	98.2
1	30	200	35	0	0.007	100
1	30	200	35	5	0.04	-
1	30	200	42.5	0	0.013	100
1	30	200	42.5	2.5	0.068	99
1	30	200	42.5	5	0.092	100
1	30	200	42.5	10	0.092	100
1	30	200	42.5	20	0.098	-
3	30	200	5	0	0.006	93.8
3	30	200	35	0	0.01	99.9
3	30	200	42.5	0	0.013	94.5
6	30	200	5	0	0.003	77.1
6	30	200	35	0	0.004	95.9
6	30	200	42.5	0	0.005	47

## Data Availability

The original contributions presented in this study are included in the article. Further inquiries can be directed to the corresponding author.
